# Group Schema Therapy for Refugees with Treatment-Resistant PTSD and Personality Pathology

**DOI:** 10.1155/2024/8552659

**Published:** 2024-02-22

**Authors:** Linda Verhaak, Jackie June ter Heide

**Affiliations:** ARQ Psychotrauma Expert Group, Diemen, Netherlands

## Abstract

**Introduction:**

Patients with complex forms of posttraumatic stress disorder (PTSD) may benefit from schema therapy. While a small number of studies point to the effectiveness of individual schema therapy in refugees with PTSD, no evidence on group schema therapy (GST) in refugees exists. To illustrate and advocate for the use of GST in refugee patients with treatment-resistant PTSD and comorbid personality pathology, a case report is presented. *Presentation*. The case concerned the treatment of an East African female refugee who survived sexual and physical violence and loss as a child, as the hostage of a rebel army, and as a victim of human trafficking. She was diagnosed with PTSD, major depressive disorder, and borderline personality disorder. Trauma-focused therapy was hampered by insufficient treatment attendance due to current stress factors and early destructive coping strategies. One year of GST enabled the patient to overcome treatment-undermining patterns and benefit from subsequent trauma-focused therapy.

**Conclusion:**

This case suggests that GST may have the potential to improve treatment adherence and the effectiveness of trauma-focused treatment in complex refugee patients. Clinical impressions need to be confirmed in a study that examines the feasibility, acceptability, and preliminary efficacy of GST in refugees with treatment-resistant PTSD and personality pathology.

## 1. Group Schema Therapy (GST) for Refugees with Treatment-Resistant PTSD and Personality Pathology

Refugees who seek treatment for trauma-related psychological distress are generally considered complex patients (e.g., [1]). This complexity is related to a range of factors, including traumatic and current stressors, the psychosocial outcomes of these stressors, and transcultural factors. Traumatic events, including persecution, war, and abuse, may take place before, during, and after migration, while daily stressors include the immigration process, financial problems, and loss of social network [2, 3]. Consequently, the psychosocial functioning of refugees may be impaired, resulting in a variety of psychological problems, including posttraumatic stress disorder (PTSD) [4], complex PTSD [5], and comorbid disorders such as depression and anxiety [6] (see Appendix A). Finally, the treatment of refugee patients may be complicated by transcultural factors, such as discrepancies in cultural beliefs about mental illness and treatment expectations [7].

Despite this complexity, psychological interventions with treatment-seeking refugees have been found to be effective [8]. Effect sizes are medium to large, with most evidence supporting trauma-focused cognitive–behavioral treatment such as narrative exposure therapy [9–11]. Between four and five refugees need to be treated in order for one to benefit [9]. In the remaining cases, additional or preparatory interventions may be necessary to enable trauma-focused therapy.

In recent years, evidence has appeared on the use of schema therapy in the treatment of patients with complicated forms of PTSD. This use is based on the understanding that schemas are central to the development and maintenance of PTSD [12, 13]. Several authors have suggested that as a result of preexisting negative core schemas, individuals can be more vulnerable to developing PTSD [14] and that their ability to process trauma-related material can be impaired [15].

Schema therapy has been developed and found effective in industrialized countries to treat patients with personality disorders. In addition, there is emerging evidence for the treatment of PTSD with schema therapy [13, 16], as well as with key elements such as Imagery Rescripting, also for refugees (ImRs) [17–20].

However, in these studies, schema therapy is employed to directly reduce PTSD symptom severity, and it may also be used for refugees who show a low response to trauma-focused treatment due to interference of rigid schemas. Some refugees, especially those with an early and long history of trauma and loss, may be susceptible to developing schemas and modes that tell the person they are worthless, that no one may be trusted, and that emotions should be avoided. Such patterns may lead to treatment-interfering behavior including avoidance of disclosure, no-show, and drop-out, which add to the complexity of treatment. In such patients, schema therapy may be employed to clarify and diminish dysfunctional schemas and modes, to consequently enable the patient to benefit from trauma-focused treatment.

While such treatments are conducted in clinical practice, no studies of schema therapy aimed at enabling subsequent trauma-focused treatment exist. To illustrate and advocate for the use of schema therapy in treatment-resistant refugees with PTSD, we here present the case of an African refugee who was offered GST to help overcome her maladaptive coping strategies and benefit from trauma-focused treatment. Through this case, we argue that despite the complexities, treating refugee patients with GST may be feasible, acceptable, and effective.

This case report was prepared following the CARE Guidelines [21]: Publication of the case was approved by the Faculty Ethical Review Board of the Faculty of Social and Behavioural Sciences, Utrecht University, the Netherlands. The patient provided written informed consent to publish her case report. The treatment took place at ARQ Centrum'45, a Dutch national center for diagnostics and treatment of patients with complex psychotrauma, in a specialized team for victims of sexual violence and exploitation.

## 2. Patient Information

### 2.1. Demographic Information and Main Symptoms

The case concerns the treatment of a female refugee from East Africa, who was referred by the mental health emergency service. At the time of referral, she was 28 years old and a single mother to a 5-year-old child. She had a residency permit but no permanent housing and was unemployed.

At intake the patient reported the following PTSD symptoms according to DSM-5: daily intrusive memories and nightmares, auditory hallucinations, episodes of dissociation with flashbacks, and intense and prolonged psychological distress and physiological reactions to internal and external triggers (intrusion cluster); avoiding distressing memories, thoughts and feelings and avoiding places and people that remind her of the past (avoidance cluster); strong negative beliefs about herself, others and the world, strong self-blame, persistent negative feelings like guilt, shame, horror, and fear, inability to experience positive emotions, diminished interest in activities she used to enjoy and a feelings of detachment and estrangement from others (negative alterations in cognitions and mood), excessive irritability and angry outbursts, constant hypervigilance, exaggerated startle response, sleep, and concentration problems (alterations in arousal and reactivity); persistent sense of unreality and detachment of herself and her surroundings (the dissociative subtype of PTSD), as well as a depressed mood and suicidal thoughts. She was diagnosed with the dissociative subtype of PTSD and with a major depressive disorder. Later in the treatment, a borderline personality disorder was classified based on emotion regulation problems, feeling empty inside, a lack of identity, low self-esteem, and fear of abandonment. Although the ICD-11 is not used in the Dutch mental health care system, the patient might be stated to suffer from a complex PTSD. She was also an intelligent, resilient, and remarkably charming woman and a loving but struggling mother to her child.

### 2.2. Personal History

The patient grew up as an only child in the east of Africa, living with her mother until the age of 8, when she witnessed her mother die in a traffic accident. After her mother's death, she was sent to a foster family, where she was neglected, mistreated, and sexually abused by her foster father. At the age of 12, she witnessed the torturing, raping, and killing of her aunt by rebels. Four years later, she herself fell victim to a rebel army, being held hostage and subjected to cruel physical and sexual violence for several years. Fleeing the rebel war, she found herself pregnant after a group rape and had to leave her first child behind in a refugee camp when she was again abducted to fight for rebels. She managed to escape the battlefront by hiding in a truck filled with corpses. At the age of 20, she arrived in the Netherlands, misled by a human trafficker who forced her to work in prostitution for 2 years, during which she endured and witnessed physical and sexual abuse, illegal abortions, and being drugged. Shortly after her escape from the trafficker, the patient became pregnant again and gave birth to a second child that she raised on her own. She enrolled at the University of Applied Sciences but was forced to quit due to concentration problems. Church and prayer were supportive to her.

## 3. Initial Treatment

At the request of the patient, treatment initially consisted of pharmacotherapy aimed at reducing the frequent auditory hallucinations. In addition, a family daycare program for patients and children was advised, and child care and social work were involved. However, the mother and child missed most appointments. Financial, housing, and integration problems, as well as underlying patterns of avoidance and distrust, were hampering the therapeutic process.

In spite of the therapeutic efforts, the patient's complaints remained severe. She experienced constant intrusive memories causing high anxiety, sleep deprivation, disgust, and despair, while at the same time struggling to be a good parent. A clinical psychologist (LV) was involved to start trauma-focused therapy, but the patient missed all initial appointments. This lasted until the therapist came for a home visit, which the patient perceived as an act of genuine interest. Additional psychoeducation about the effect of trauma on the brain and the potential positive effects of EMDR therapy motivated the patient to give trauma-focused treatment a try because, as she said, she owed it to her child.

In order to relieve her auditory hallucinations of crying babies, the patient started with EMDR focused on processing the loss of her first child. Shortly after, the patient became pregnant with twins, which resulted in a stillbirth. This caused the patient to totally withdraw from treatment for months. Subsequently, EMDR was resumed, focusing on the stillbirth and associated feelings of guilt and worthlessness. However, psychosocial problems, such as conflicts with her debt counselor, the assignment of a child protection guardian, and somatic complaints, slowed down the therapy process considerably.

In addition, old emotional and behavioral patterns that the patient had developed to deal with loss and trauma from a young age continued to undermine treatment. Nightmares, compelling voices, or external adversities could cause the patient to withdraw to her bedroom for days on end, distrusting and avoiding contact with everyone, including her child, hating herself, and feeling desperately lonely and suicidal.

GST was indicated to help the patient learn to recognize and change her persistent survival modes and internalized critical voices, and begin to understand and grow her healthy adult mode, strength, and resilience. Working in a group was favored over individual therapy to promote a feeling of belonging and corrective emotional learning. A secondary aim was to motivate her for trauma-focused treatment with less avoidance.

## 4. GST

Treatment consisted of GST based on the mode model by Farrell and Shaw [22, 23]. It consisted of 40 weekly group sessions, lasting 90 min, over a 1-year period. GST was offered in a day treatment format, which further consisted of group psychomotor therapy and an optional individual appointment. The group was conducted in English and included six refugee women from different countries who all suffered from PTSD and personality pathology. The GST was run by two certified schema therapists (including LV).

GST starts with psychoeducation about the mode model, describing schema modes as emotional and behavioral states that can be grouped into adaptive modes (happy child and healthy adult) and maladaptive modes (child, coping, and parent modes), originating at a young age to emotionally manage negative life events. To allow for the growth of emotional awareness and emotional tolerance, mindfulness exercises are incorporated. Group members learn to identify their unmet emotional needs in childhood, their ways of dealing with difficult emotions, and healthy ways to get their needs met. Experiential and interpersonal techniques such as limited reparenting, chair work, and empathic confrontation are employed from an early stage. Connection between and recognition among the group members is promoted.

## 5. Treatment Course

Before starting GST, the patient completed two schema therapy measures. Maladaptive schemas were assessed using the Young Schema Questionnaire [24]. The patient had clinically meaningful scores on all schemas except Entitlement and Enmeshment. Modes were assessed using the Schema Mode Inventory [25]. The patient had elevated (>60%) scores on all maladaptive modes, especially on compliant surrender (91%) and detached protector (80%), demanding parent (77%), vulnerable child (88%), and a very low score on the adaptive happy child mode (6%). In addition, the patient was asked to complete ROM assessments, but her compliance was very low. The assessments triggered memories that made her angry and sad, causing her to cancel appointments for weeks on end.

Despite her reservations about group treatment, the patient agreed to participate in GST. In the beginning, it felt unreal and strange to be treated in a friendly and caring manner, and she expected that she would soon have to return some unwanted favor. In the group, she experienced nervousness and increased internal voices. She responded by being active and talkative, which was recognized as a protector mode that helped her stay away from her feelings of vulnerability and anxiety but also caused emotional distance and prevented genuine connection. After 2 months, she opened up to the other group members, found recognition, and reported a sense of belonging and less loneliness.

Nevertheless, she kept canceling GST sessions or was late due to all kinds of stressors. Consequently, a rule was introduced that she could miss or be late for a maximum of one appointment per month. On the one hand, this motivated the patient to prioritize the GST appointments; on the other hand, she interpreted this as a lack of understanding of her vulnerability and the complexity of her situation. Referring back not only to the basic needs of love, understanding, and support but also to setting realistic boundaries helped her accept and understand.

Throughout the treatment, her social situation remained hectic. Halfway into the GST, the patient received news that her firstborn child, whom she had been searching for, had been found. This lead to joy as well as to emotional and practical worries associated with potential family reunification. Shortly after, her second child expressed a wish to be in contact with her biological father, which was very stressful to her. Around the same time, she applied for naturalization and a Dutch passport, resulting in financial stress and anxiety about authorities finding out that she had worked with the rebels.

In GST, the focus was on understanding the origin and function of persistent protector modes and demanding/punishing parent modes, creating language, and ways to deal with the protectors and the child modes, and improving the therapeutic relationship and trust. The patient recognized that the greater her protector (not wanting to attend or to withdraw), the greater the needs of her vulnerable child (sadness, loneliness). She found the experiential exercises in the group emotionally overwhelming yet helpful. Imaginary Rescripting (ImRs) and relaxation exercises were recorded on the patient's mobile phone, and she reported the calming effect of listening to these at home.

Following 1 year of GST, trauma therapy, including EMDR and ImRs, was resumed, focusing on deeply shameful events that the patient had never shared before, like early sexual abuse and atrocities during her time in forced prostitution. Life events still caused many no-shows. However, when present, she was more compliant and less avoidant during the trauma sessions, due to a better understanding of her avoidance tendencies. In the ImRs, the therapist cared for the child and spoke up to the perpetrators; during EMDR therapy, the therapist helped the patient find new, more compassionate conclusions about herself.

## 6. Outcome

After treatment, most PTSD complaints were in remission. The first positive changes in symptoms were noticeable in the intrusion cluster, including the diminishing of hearing voices, nightmares, flashbacks, and dissociative episodes. After 6 months in GST, the patient reported less avoidance of certain situations and people that reminded her of the past (avoidance cluster); she reported less overwhelming negative emotions, became less anxious of her own emotions, started to make more contact with others, even with her ex-partner and his partner, reported experiencing more positive emotions together with her daughter and more interest in activities (diminishing negative alterations in cognitions and mood), reported less irritability and angry outbursts, allowing her to become more patient with her daughter (hyperarousal cluster). The remaining PTSD symptoms included auditory hallucinations and nightmares, although less frequent, and concentration problems.

The patient had also accomplished large improvements in the area of her personality problems. Her ability to regulate her emotions grew, and she learnt to subtitle her moods and needs, which in turn had a positive effect on her relationship with her child, whereas at the start of therapy, the patient hardly came out of her house, she was now much more capable to take care of the household and go out for outdoor activities with her daughter. Her self-esteem grew, and she no longer felt like a “nobody,” as she started an online group for women of her nationality to share worries, help each other and pray together. Her distrust diminished, as she became better able to understand the behavior of others, resulting in better social relationships. The depression was in remission at the end of treatment, as the patient reported less hopelessness and negative thinking, had more interest and pleasure in activities, more energy, and less feelings of worthlessness.

Finishing the therapy was a difficult step that the patient did not feel ready to take. The farewell session never took place despite several attempts to reschedule. Two months later, she sent an email to share that she enrolled in university and wished to help others.

## 7. Patient Perspective

During treatment, a shared interest to document the patient's treatment process emerged. After completing her treatment, the patient agreed on the publication of a case report. The manuscript was presented to her for comments and approval before submission.

Shortly after the GST, she was interviewed: “I'm glad to say that I'm almost like a mini therapist to myself, I recognize my modes (..) and I relate them to my day-to-day life. They have helped me recognize and be more alert of myself, I'm not more of self-destruction to myself like I was before. It helped me more to care of myself, to care of that little child that was never taken care of. When you are bombarded with such difficulties in life, that is actually when you need schema therapy the most. This healthy adult is even helping me with the voices. (In the group) we recognized we had the same problems. Then different backgrounds don't matter.”

## 8. Discussion

In this case report, the treatment of a multiply traumatized, treatment-resistant refugee patient struggling with current stressors and destructive patterns is described. GST was initiated to treat her personality problems and to facilitate trauma-focused therapy. Through GST, the patient was able to break the vicious cycle of avoidance of negative emotions and engage in further trauma-focused treatment.

The case implies that treating refugee patients with GST seems possible in spite of challenges in the areas of attendance, language, transcultural differences, and complicating postmigration factors. This is in line with a growing body of research that suggests that refugee patients may benefit from ImRs [17, 19, 20]. This case suggests that schema therapy offered in a group may also be feasible and effective. Even more so than individual schema therapy, GST with refugees poses transcultural challenges as patients and therapists with diverse linguistic, ethnic, and cultural backgrounds need to work together. While explanations of illness among refugee patients and therapists may differ [7], GST seems able to find a common language that helps participants work on their psychological recovery (see [26]).

While GST may be indicated for refugees who present with PTSD and comorbid personality pathology, diagnosing personality problems in refugee populations presents a special challenge. Diagnostic assessment must be culturally informed to avoid the risk of under- or overdiagnosing [27]. In this case, the assessment of the patient's personality pathology was carefully weighed in the perspective of her migration process and cultural dimensions. In refugee patients, childhood trauma-related symptomatology, such as emotion-regulation difficulties, interpersonal problems, impulsive behavior, and dissociation, may be labeled as complex PTSD [5].

Complex PTSD calls for trauma-focused treatment. However, additionally labeling the complex symptoms as personality pathology may open up additional treatment options and research venues. In studies of Western populations, personality disorders are associated with high rates of treatment noncompletion [28]. In refugee populations, such research is lacking, as most studies focus mainly on PTSD and depression [29]. The core feature of emotional dysregulation is a shared symptom in both complex PTSD and borderline personality disorder [30].

To determine the feasibility, acceptability, and efficacy of GST for refugees with treatment-resistant PTSD and personality pathology, research is needed. However, conducting research on this population presents various challenges. While repeated measures may be challenging to any patient, diagnostic instruments that have not been specifically designed for refugee patients and that have been translated or are interpreted during administration may be extra burdensome (e.g., [31]). This stresses the need for multi-method research that includes quantitative measurements as well as qualitative interviews that enable refugee patients to voice their own experiences and opinions. In addition, low attendance and risk of dropout due to rigid treatment-interfering patterns and concurrent postmigration stressors may limit the feasibility of research with this population. As illustrated in this case, the lives of refugee patients, especially those submitted to human trafficking, may be extremely eventful (e.g., [32]). Concurrently addressing postmigration stressors is essential to enable refugees to benefit from GST. Any study design would have to take this into account.

In conclusion, this case shows the potential of add-on GST for chronic PTSD in complex refugee patients: those who suffered early childhood adversities, live in complicated postmigration circumstances and benefit insufficiently from trauma-focused treatment due to destructive coping patterns. GST may be able to offer a common language that enables patients to benefit from further trauma-focused treatment, thus opening new roads to recovery.

## Data Availability

The access is restricted due to legal and ethical concerns, such as patient privacy and confidentiality.

## Appendix

### A. Posttraumatic stress disorder in refugees

The diagnosis of posttraumatic stress disorder (PTSD) is included in both the 5th edition of the Diagnostic and Statistical Manual of Mental Disorders (DSM-5) and the 11th edition of the International Classification of Diseases (ICD-11). In DSM-5, PTSD is defined by 20 symptoms grouped into four main symptom clusters, i.e., intrusion symptoms, avoidance of stimuli, negative alterations in cognitions and mood, and alterations in arousal and reactivity. Patients may also suffer from PTSD of the dissociative subtype (PTSD-DS), which includes depersonalization and/or derealization in addition to the main symptom clusters and generally refers to a more severe or complex form of PTSD. Refugees have generally high rates of PTSD [33]. Meta-analytically, the prevalence of DSM-5 PTSD in refugees is estimated at around 30% [34]. The presence of PTSD-DS in refugees has been estimated to be around 29% ([35], in preparation). In ICD-11, the PTSD diagnosis is limited to reexperiencing, avoidance, and heightened perceptions of current threats. In addition, patients may be diagnosed with complex PTSD (CPTSD), which includes the main PTSD symptoms as well as disturbances in three domains of self-organization: affective dysregulation, negative self-concept, and disturbances in relationships. ICD-11 PTSD and CPTSD may not be diagnosed simultaneously. The prevalence of CPTSD in refugees ranges between 16% and 38% in treatment-seeking samples and between 2% and 51% in population samples [5].
